# Detection of *Chaetomium globosum*, *Ch. cochliodes* and *Ch. rectangulare* during the Diversity Tracking of Mycotoxin-Producing *Chaetomium*-like Isolates Obtained in Buildings in Finland

**DOI:** 10.3390/toxins12070443

**Published:** 2020-07-08

**Authors:** Johanna M. Salo, Orsolya Kedves, Raimo Mikkola, László Kredics, Maria A. Andersson, Jarek Kurnitski, Heidi Salonen

**Affiliations:** 1 Department of Civil Engineering, Aalto University, Box 12100, FI-00076 Aalto, Finland; raimo.mikkola@aalto.fi (R.M.); aino.andersson@aalto.fi (M.A.A.); jarek.kurnitski@aalto.fi (J.K.); heidi.salonen@aalto.fi (H.S.); 2 Department of Microbiology, Faculty of Science and Informatics, University of Szeged, Közép fasor 52, 6726 Szeged, Hungary; varga_orsi91@yahoo.com (O.K.); kredics@bio.u-szeged.hu (L.K.); 3 Department of Civil Engineering and Architecture, Tallinn University of Technology, Ehitajate tee 5, 19086 Tallinn, Estonia

**Keywords:** *Chaetomium globosum*, Chaetomium cochliodes, *Chaetomium rectangulare*, mycotoxin, chaetoglobosin, chaetomin, chaetoviridin A and C, chaetomugilin D, indoor mold, fluorescence

## Abstract

The diversity of *Chaetomium-*like isolates in buildings in Finland is poorly documented. This paper describes a set of methods for rapid diversity tracking of 42 indoor *Chaetomium*-like isolates. These isolates were categorized based on their fluorescence emission, ascomatal hair morphology, responses in three bioassays and resistance/sensitivity to the wetting agent Genapol X-080. Thirty-nine toxigenic isolates were identified [*Ch. globosum* (*n* = 35), *Ch. cochliodes* (*n* = 2) and *Ch. rectangulare* (*n* = 2)]. These isolates were identified down to the species level by *tef1α* gene sequencing. The major toxic substances in the ethanol extracts of the *Ch. globosum* and *Ch. cochliodes* strains were chaetoglobosin, chaetoviridin A and C, chaetomugilin D and chaetomin, identified based on HPLC-UV and mass spectrometry data (MS and MS/MS). Ethanol extracts from pure *Ch. globosum* cultures exhibited a toxicological profile in the boar sperm motility inhibition assay (BSMI), sperm membrane integrity damage assay (SMID) and inhibition of cell proliferation (ICP) assay, similar to that exhibited by pure chaetoglobosin A. Overall, differences in fluorescence, morphology, toxicity profile, mycotoxin production and sensitivity to chemicals were consistent with those in *tef1α* sequencing results for species identification. The results indicate the presence of *Ch. cochliodes* and *Ch. rectangulare* in Finnish buildings, representing a new finding.

## 1. Introduction

The family *Chaetomiaceae* consists of globally ubiquitous fungal genera that are found in soil and degraded cellulosic materials, such as dung and wastes. This family comprises mesophilic, thermotolerant and thermophilic genera which demonstrate mycelial growth at 15−55 °C. The genera belonging to *Chaetomiaceae* are characterized by ascomata containing ascospores in deliquescent asci found inside the ornamented perithecia [1,2,3]. Indoor isolates producing dark pigmented hairy ascomata are usually assigned to the genus *Chaetomium* and are often tentatively recognized at the species level as *Chaetomium globosum* or *Chaetomium* spp., which has been reported as a dominant fungal species in damp and water-damaged building materials in Denmark and Greenland [4,5].

A member of *Chaetomiaceae, Ch. globosum* is the most common if not the only member of this family to be found in indoor environments in Finland [6,7,8]. It is also the only species and a member of the only genus in *Chaetomiaceae* to be listed in the Finnish Environmental Relative Moldiness Index (FERMI). This simplified metric based on 10 mold species and the inclusion of these 10 species into the FERMI were explained by Täubel et al. [8]. However, not all dark ascomata-producing isolates found in indoor environments are *Ch. globosum*, and they are not necessarily members of the genus *Chaetomium.* A study involving 145 indoor *Chaetomiaceae* isolates identified in 19 countries reported that 30 species found in indoor environments could be accommodated in 10 genera of *Chaetomiaceae* [2]. The most common indoor isolates worldwide belong to the genus *Chaetomium*, and they were assigned to the *Ch. globosum* complex and to *Ch. globosum, Ch. cochliodes* and *Ch. elatus* (closely related to *Ch. rectangulare*); collectively, they represent 70% of the 145 isolates [2,3,9]. These studies showed that, although the members of the *Ch. globosum* complex were the most abundant in indoor environments, other *Chaetomium*-like isolates were also present. The high number of newly detected species and genera indicated that the diversity of indoor *Chaetomiaceae* is not well known [3]. 

Mycotoxins are bioactive secondary metabolites excreted by fungi. They regulate metabolic and ecological processes essential for a producer [10]. In mammalian cells, they may interfere with cellular structures (e.g., biomembranes) and with important cellular processes (e.g., protein, RNA and DNA syntheses; ion homeostasis; energy metabolism; and mitochondrial functions) [11,12,13,14,15,16,17,18]. Most mycotoxins exert immunosuppressive effects, and many of them are cytotoxic/cytostatic and thus could potentially damage the skin and lungs and could affect the gut microbiota [19,20,21,22]. In addition, they may affect the physical defense mechanisms of the respiratory tract, and this phenomenon is described as the ciliostatic effect of the metabolites produced by *Chaetomium* spp. Ciliostatic effects may reduce the ability of the respiratory tract to purify particulate pollutants (including bacteria or viruses) [20,23,24,25]. Moreover, mycotoxins could damage the macrophages of alveoli, thereby preventing the elimination of pollutants from the deeper lungs, resulting in increased susceptibility to infectious diseases and in reduced defense against other contaminants [19,23,24,25,26,27,28]. However, the health risk associated with mycotoxins in urban indoor environments remains controversial [21], and no safety limits for exposure to airborne mycotoxins or to indoor mold growth have been established.

The presence of the representants of *Chaetomiaceae*, especially the representants of the genus *Chaetomium,* in indoor environments is of concern due to the following reasons: (A) The representants of the genus *Chaetomium* degrade moist cellulose-containing building materials, such as timber and plywood, as well as synthetic building materials, such as plastics. In moist materials, they may evolve as the dominant fungal colonizers, forming dense mycelial growths covering large areas of building materials, thereby possibly affecting building structures [5,29]. (B) A causal relationship between exposure to actively growing indoor molds (identified based on mold odor and visible mold growth on building materials) and the development and exacerbation of asthma in children is suggested in a review of longitudinal studies [30]. The secondary metabolites of the genus *Chaetomium* are suspected to exert a negative impact on human health. The secondary metabolites of *Ch. globosum*, such as chaetoglobosins and chaetoviridins/ chaetomugilins, have been found in building materials in quantities of 950 and 200 mg m^−2^, respectively [9]. (C) Representants of the genus *Chaetomium* are associated with severe secondary infections in human patients and also with onychomycoses in healthy individuals [31]. In addition, members of the genus *Chaetomium* may have a latent clinical potential to cause invasive mold infections [32]. However, whether the species constituting the *Ch. globosum* complex, which cannot grow at temperatures higher than 35 °C, are causing the described infections remains controversial. The reported infections may have been caused by closely related species included in the *Ch. globosum* complex, such as *Ch. megalosporum* [3,32,33].

The negative impacts of *Chaetomium* infestation in buildings have most often been associated with *Ch. globosum*. Such a correlation justifies the inclusion of *Ch. globosum* as an indicator fungus in the FERMI; at the same time, it underlines the importance of accurate identification of the species and the acquisition of knowledge on the diversity of *Chaetomium*-like fungi in indoor environments in Finland. Recognition of toxic metabolite-producing *Chaetomium* isolates, especially the *Ch. globosum* isolates, is thus of concern, and methods that speed up the diversity tracking of indoor *Chaetomiaceae* isolates may be useful for the rapid characterization of fungal growth on building materials.

In this study, a set of methods was used in the diversity tracking of 42 toxigenic indoor *Chaetomiaceae* isolates found in buildings in Finland. The methods identified three species that constitute the *Chaetomium globosum* complex: *Ch. globosum, Ch. cochliodes* and *Ch. rectangulare*. Moreover, the mycotoxins produced by *Ch. globosum* and *Ch. cochliodes* were identified, and their biological activities were compared by cell-based bioassays. The species-specific sensitivity of the indoor *Chaetomium* isolates to indoor chemicals and biocides were also described.

## 2. Results

### 2.1. Screening for Three Toxigenic Indoor Chaetomium-Like Isolates: Ch. globosum, Ch. cochliodes and Ch. rectangulare

**Figure 1:**
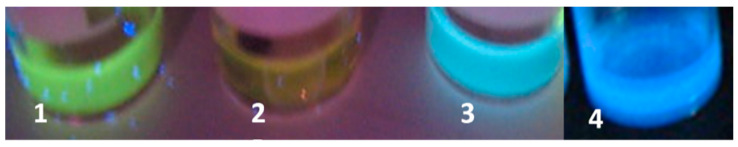
Fluorescence emissions of the biomass suspensions of selected *Chaetomium*-like isolates excited with UV light at 360 nm: (**1**) *Chaetomium rectangulare* MO15; (**2**) *Ch. cochliodes* OT7; (**3**) *Ch. globosum* HAS5; (**4**) Unidentified *Chaetomium*-like isolate Ch1/tu; and (F) *Ch. cochliodes* OT7b.

A combination of three screening methods was employed to detect cultivable toxigenic *Chaetomium* isolates from indoor dusts collected from eight problematic buildings in Finland. Single colonies (*n* = 220) picked from the cultivation plates were inspected for: (1) Toxicity of biomass suspensions in two bioassays: The results of the assays revealed that 130 colonies out of the 220 were screened to be toxic. (2) Production of ascomata: Inspection under a stereomicroscope and phase contrast microscope revealed that 42 colonies out of the 130 toxigenic colonies produced ascomata. (3) Fluorescence emitted by the biomass suspensions: When exitated with UV-light, biomass suspensions prepared from the 42 toxigenic ascomata-producing colonies emitted blue-green, blue, yellow-green or no fluorescence, as shown in Figure 1.

**Figure 2:**
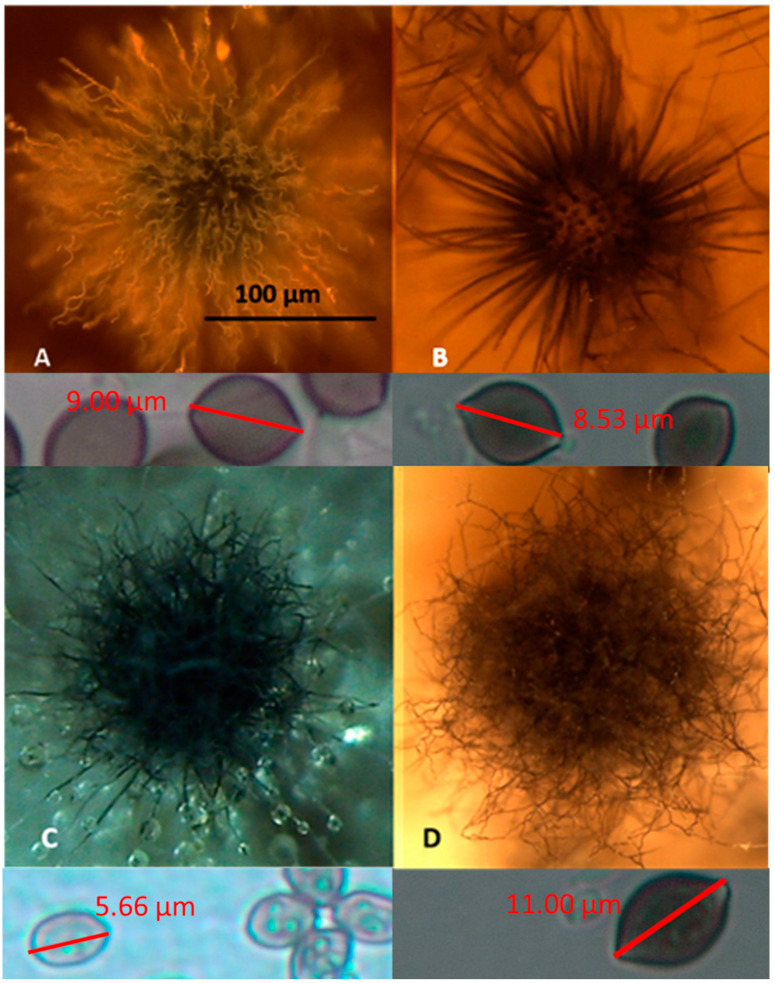
Stereomicrographs of the four morphotypes (A–D) exhibited by the 42 toxigenic *Chaetomium-*like isolates. (**A**) The *Ch. globosum* MTAV35 strain displayed a morphotype characterized by a curly ascomatal hair, and it produced 9 µm × 10 µm globous ascospores. (**B**) The *Ch. cochliodes* OT7 strain exhibited a straight ascomatal hair and produced 8 µm × 9 µm oval ascospores. (**C**) The unidentified *Chaetomium*-like strain Ch1/tu C exhibited straight, dichotomously branched ascomatal hair and produced 4 µm × 6 µm oval ascospores. (**D**) The *Ch. rectangulare* MO15 strain exhibited a curly, dichotomously branched ascomatal hair and produced 6–7.5 µm × 10–11 µm elongated ascospores.

The 42 colonies characterized as toxigenic *Chaetomium*-like isolates were pure-cultured and categorized into four morphotypes based on the following: fluorescence emission of their biomass suspensions (Figure 1), morphology of their ascomata and size and shape of their ascospores (Figure 2). The strain codes, origin, sampling sites, morphotypes and fluorescence emissions, as well as the identified species and genera, are presented in Table 1.

Representative strains exhibiting the four different morphotypes (A–D) were identified down to the species/genus level (Table 1) as follows: (A) *Ch. globosum* exhibits a coiled, unbranched ascomatal hair, large globous ascospores and biomass suspension emitting blue-green fluorescence; (B) *Ch. cochliodes* exhibits straight, unbranched ascomatal hair, large oval ascospores and biomass suspensions emitting no fluorescence; (C) the unidentified *Chaetomium*-like isolates exhibit dichotomously branched ascomatal hair, small elongated ascospores and biomass suspensions emitting blue fluorescence; and (D) *Ch. rectangulare* exhibits branched ascomatal hair, large elongated ascospores and biomass suspensions emitting yellow-green fluorescence. Eleven isolates displaying the morphotypes A, B and D were very toxic in the BSMI and ICP assays, and they were identified as members of the *Ch. globosum* complex. Seven isolates obtained from seven buildings and displaying the morphotype A were identified as *Ch. globosum*. Two isolates displaying the morphotype B (OT7 and OT7b) and two other isolates displaying the morphotype D were identified as *Ch. cochliodes* and *Ch. rectangulare*, respectively. Strains Ch1/tu, Ch2/tu, Ch3/tu and Ch4/tu with morphotype C were only slightly toxic in the BSMI assay, nontoxic in the ICP assay and differed from the strains belonging to the *Ch. globosum* complex based on their small-sized ascospores. These strains were isolated from an inlet air filter and possibly came from the outdoor air (Table 1).

### 2.2. Mycotoxins in the Fungal Extracts

**Figure 3:**
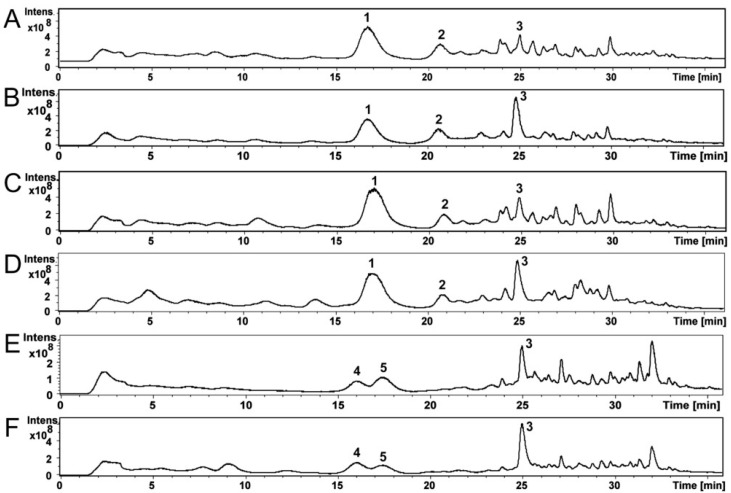
The HPLC-MS total ion chromatograms of the ethanol extracts of *Ch. globosum* strains MTAV35 (**A**), HAS5 (**B**), RUK10 (**C**) and ABCD (**D**) and *Ch. cochliodes* strains OT7 (**E**) and OT7b (**F**) and their compounds **1**–**5**.

Compounds of the *Ch. globosum* and *Ch. cochliodes* ethanol extracts were identified using high-performance liquid chromatography–mass spectrometry (HPLC-MS). The HPLC-MS total ion chromatograms of the ethanol extracts of *Ch. globosum* and *Ch. cochliodes* and their compounds are shown in Figure 3A–F. The *Ch. globosum* strains (Figure 3A–D) total ion chromatograms highly resemble each other as well as the *Ch. cochliodes* strains (Figure 3E,F). 

Figure S1A–F shows MS and MS/MS spectra of *Ch. globosum* RUK10 compounds **1**–**3** (Figure 3C). Compound **1** (Figure 3C) at 17.0 min of *Ch. globosum* RUK10 contained a protonated mass ion [M + H]^+^ at *m/z* 529.4, a sodiated mass ion [M + Na]^+^ at *m/z* 551.3 and a mass ion of dimer [2M + Na]^+^ at m/z 1079.4 of chaetoglobosins (Figure S1A). The MS/MS spectrum of the precursor mass ion at *m/z* 529.9 produced mass ions at *m/z* 511.3, 457.2 and 400.3 (Figure S1B), as reported by Xu et al. [35] for chaetoglobosin C. The diagnostic fragmentation ion at *m/z* 184.9 indicated a tryptophan-containing moiety of chaetoglobosins (Figure S1B), as reported by Walsh et al. [36]. Compound **1** UV spectrum had maximum wavelengths at 220 and 280 nm (Figure S2A), similar to as reported for chaetoglobosin A [37]. However, compound **1** could not be identified to any specific member of chaetoglobosins family and therefore it was named as chaetoglobosin. Compound **2** of *Ch. globosum* RUK10 (Figure 3C) at 20.8 min contained a protonated mass ion [M + H]^+^ at *m/z* 435.2 and a sodiated mass ion [M + Na]^+^ at *m/z* 457.2 and a mass ion of dimer [2M + Na]^+^ at *m/z* 891.3 of chaetoviridin C (Figure S1C). The MS/MS spectrum of the precursor mass ion at *m/z* 435.8 produced mass ions at *m/z* 417.1, 389.1 and 316.1 (Figure S1D). Chaetoviridin C had maximum wavelengths at 225, 294 and 390 nm (Figure S2B). Compound **3** of *Ch. globosum* RUK10 (Figure 3C) at 24.9 min contained a protonated mass ion [M + H]^+^ at *m/z* 433.2, a sodiated mass ion [M + Na]^+^ at *m/z* 455.2 and a mass ion of dimer [2M + Na]^+^ at *m/z* 887.3 of chaetoviridin A (Figure S1E). The MS/MS spectrum of the precursor mass ion at *m/z* 433.9 produced the mass ions at *m/z* 333.1 and 389.1 (Figure S1F), indicating the fragmentation of chaetoviridin A, as also reported by Larsen et al. [38]. Compound **3** also had similar UV spectrum, having maximum wavelengths at 228, 310 and 366 nm (Figure S2C), as reported for chaetoviridin A [38]. Other *Ch. globosum* strains MTAV35, HAS5 and ABCD contained compounds **1**–**3** at same retention time (Figure 3A,B,D) and had similar MS, MS/MS and UV spectra as described above for the *Ch. globosum* RUK10.

Compound **4** of the *Ch. cochliodes* OT7 (Figure 3E) at 16.0 min contained a protonated mass ion [M + H]^+^ at *m/z* 435.2 and a sodiated mass ion [M + Na]^+^ at *m/z* 457.2 and a mass ion of dimer [2M + Na]^+^ at *m*/*z* 891.2 of chaetomugilin D (Figure S1G). The MS/MS spectrum of the precursor mass ion at *m/z* 435.9 produced the mass ions at *m/z* 417.2, 389.2 and 361.1 (Figure S1H). Compound **4** had UV spectrum with the maximum wavelengths at 220, 295 and 386 nm (Figure S2D), similar to as reported for chaetomugilin D by McMullin et al. [39]. Compound **5** of the *Ch. cochliodes* OT7 (Figure 3E) at 17.4 min contained a protonated mass ion [M + H]^+^ at *m/z* 711.2, a sodiated mass ion [M + Na]^+^ at *m/z* 733.2 and [M-S2+Na]^+^ at *m/z* 669.3 and a mass ion of dimer [2M + Na]^+^ at *m/z* 1443.0 of chaetomin (Figure S1I). The MS/MS spectrum of the precursor mass ion at *m/z* 711.7 produced the mass ion at *m/z* 645.2 indicating neutral loss of H_2_S_2_ producing the mass ion [M − H_2_S_2_ + H]^+^ (Figure S1J). A complementary pair of fragment ions of the precursor mass ion at *m/z* 711.7 were found at *m/z* 348.0 and 364.1. The fragment ion at *m/z* 298.0 represent loss of a HSSH of mass ion *m/z* 364.0 and fragment ion at *m/z* 282.0 represent loss of HSSH of mass ion *m/z* 348.0, as also reported by Wu et al. [40] for chaetomin. UV spectrum of chaetomin had maximum wavelengths at 220, 275 and 285 nm (Figure S2E). Compound **3**, chaetoviridin A, which was also found from *Ch. globosum* strains, was detected in *Ch. cochliodes* OT7 (Figure 3E) at 24.9 min and contained a protonated mass ion [M + H]^+^ at *m/z* 433.2, a sodiated mass ion [M + Na]^+^ at *m/z* 455.2 and a mass ion of dimer [2M + Na]^+^ at m/z 887.3 (Figure S1K). The MS/MS spectrum of the precursor mass ion at *m/z* 433.8 produced the mass ions at *m/z* 333.0 and 389.1 (Figure S1L). The *Ch. cochliodes* OT7b also contained compounds **3**–**5** at the same retention time (Figure 3F) and had similar MS, MS/MS and UV spectra as described above for *Ch. cochliodes* OT7. 

Based on retention times, UV spectra, MS and MS/MS mass spectrometric data and the earlier published UV spectra and mass spectrometric data, compounds **1**–**3** of the *Ch. globosum* strains MTAV35, HAS5, RUK10 and ABCD were identified as chaetoglobosin and chaetoviridin C and A, respectively, while compounds **3**–**5** of the *Ch. cochliodes* strains OT7 and OT7b were identified as chaetomugilin D, chaetomin and chaetoviridin A, respectively (Table 2). The amounts of mycotoxins present in the ethanol extracts were calculated from the total absorbance (220 nm) of the HPLC-UV chromatograms (Table 2).

### 2.3. Mycotoxin and Toxicity Profiles of the Ch. globosum and Ch. cochliodes Isolates

Ethanol extracts containing 10 mg ethanol-soluble dry substances dissolved in 1 mL ethanol were prepared from plate-grown biomass (wet wt, ca. 200–400 mg) of the selected *Ch. globosum* and *Ch. cochliodes* strains. The ethanol extracts of the *Ch. globosum* strains emitted green fluorescence, similar to the biomass lysates. On the contrary, no fluorescence was observed from the extracts of the *Ch. cochliodes* strains. The chemical analyses (Table 3) indicated that the plate-grown biomass of the *Ch. globosum* strains contained ca. 1% of chaetoglobosin, whereas the chaetomin concentration was only 0.25% of the collected biomass. 

The biological activities of the ethanol extracts of the indoor *Ch. globosum* and *Ch. cochliodes* strains were indicated by their toxicological profiles obtained by the three bioassays. These bioassays measured the sublethal (BSMI_M_) and lethal (SMIDA_M_) toxicities in exposed motile spermatozoa, as well as the cytostatic toxicity (ICP) using continuous (PK-15) and malignant (murine neuroblastoma [MNA]) cell lines (Table 3).

The tested ethanol extracts of the *Chaetomium* strains exhibited two toxicity profiles based on the differences in EC_50_ concentrations of the ethanol-soluble dry solids obtained by the three assays (Table 2). The ethanol extracts of Group I—consisting of the *Ch. globosum* strains MTAV35, HAS5, RUK10 and ABCD—contained high concentrations of chaetoglobosin at 4 mg mL^−1^ (>85% *w*/*v* of the identified mycotoxins), but chaetomin was not detected. The extracts in Group I showed similar EC_50_ concentrations in the sublethal BSMI_M_ and in the ICP assays—5–10 µg mL^−1^—which is 100 times lower than the EC_50_ concentrations in the lethal SMID_M_ assay (300–450 µg mL^−1^). This toxicity profile indicated that the sublethal and cytostatic toxicities were similarly strong, and it was consistent with the toxicity profile of pure chaetoglobosin A (but it differed from that of the lethal toxin alamethicin and the sublethal mitochondrial toxin valinomycin). 

In Group II, the extracts obtained from the *Ch. cochliodes* strains OT7 and OT7b contained high chaetomin concentrations (1 mg mL^−1^; >70% *w*/*v* of the identified mycotoxins), whereas chaetoglobosin and chaetoviridins C were undetectable and chaetoviridin A and chaetomugilin D were detected at low concentration (0.02–0.3 mg mL^−1^). These extracts exhibited the highest toxicity in the ICP assays, with an EC_50_ of <1 µg mL^−1^. This result indicates a strong cytostatic activity similar to that exhibited by pure sterigmatocystin and citrinin (Table 2). Moreover, the extracts of OT7 and OT7b exhibited sublethal motility-inhibiting effect on the exposed spermatozoa. The sublethal activity was indicated by the 30–50 times lower EC_50_ concentration in the BSMI_M_ assay (10 µg mL^−1^) compared with the EC_50_ concentrations of 300–500 µg mL^−1^ in the SMID_M_ assay that indicated lethal toxicity.

Chaetoviridins A and C and chaetomugilin D were detected in low amounts (3–15%) in the ethanol-soluble extracts of each of the *Chaetomium* strains screened for toxicity (Table 3). Therefore, the main difference between Groups I and II was the presence of either chaetoglobosin or chaetomin in their ethanol-soluble extracts. The extract from strain RUK10 containing the smallest amounts of chaetoviridins (<0.09 mg mL^−1^) exhibited the smallest EC_50_ concentration (i.e., the highest toxicity in the BSMI and ICP assays among the tested *Ch. globosum* strains). Considering these results, we hypothesized that the chaetoglobosins were the main cause of the sublethal sperm motility inhibition and of the cytostatic effect of the *Ch. globosum* ethanol extract. We also hypothesized that chaetomin in the *Ch. cochliodes* extracts caused a considerably strong cytostatic toxicity (as indicated by the PK-15 and MNA cell proliferation) and that chaetomin exposure caused the motility-inhibiting activity in the exposed sperm cells.

**Figure 4:**
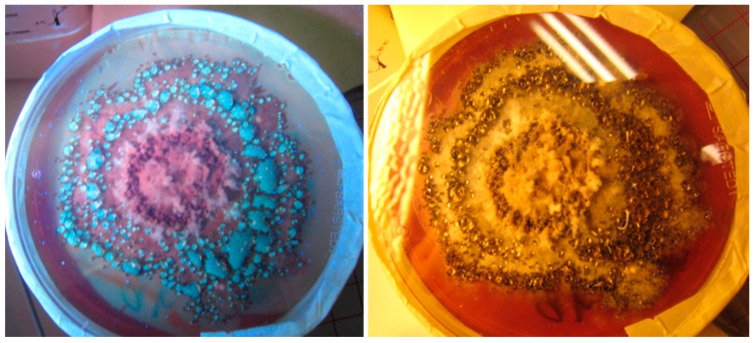
Pure culture of *Chaetomium globosum* imaged under UV (**left**) and visible light (**right**). Strain MO9 was grown on malt extract agar (MEA) at 24 °C for two weeks. Exudate-containing vesicles emitting blue-green fluorescence are shown in the left panel.

The exudate of *Ch. globosum* 2c/MT (Table 3) emitted blue-green fluorescence and was found to contain chaetoglobosin. Moreover, the fluorescent exudates of MO9 (Figure 4) were found toxic in bioassays. Based on these results, we hypothesized that the chaetoglobosin produced by strain 2c/MT and the toxin produced by strain MO9 may be excreted as liquid exudates.

### 2.4. Characterization of the Toxicological Profile of Pure Chaetoglobosin A and Chaetoglobosin-Containing Extracts

**Figure 5:**
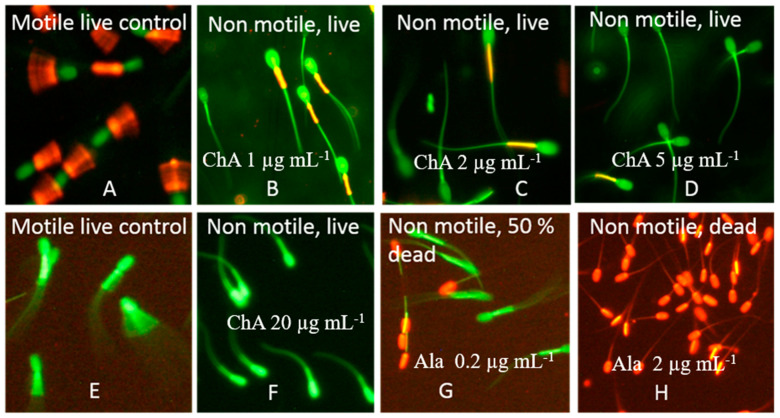
Fluorescence micrographs showing the test results for mitochondrial membrane potential ΔΨ_m_ and SMID_R_ assay. Boar spermatozoa were exposed to chaetoglobosin A (ChA) for two days at 24 °C. The sperm cells were stained with the membrane potentiometric dye JC-1 (top) and with the live–dead stain calcein-AM plus propidium iodide (PI) (bottom). The motile sperm cells in (**A**) and (**E**) were exposed to 1% ethanol as negative controls. (**B**–**D**,**F**) The sperm cells immobilized by ChA. The sperm cells in (**A**) exhibit high ΔΨ_m_ as indicated by the orange emission in the midpiece of the sperm flagellum; the green-staining sperm cells in (**E**) exhibit intact plasma membranes impermeable to the red stain PI. The red-staining sperm cells have lost their plasma membrane integrity and became permeable to PI. The sperm cells in (**G**,**H**) (positive controls for lethal toxicity) were exposed to alamethicin (Ala).

The long-term effects of exposure to chaetoglobosin A and to the chaetoglobosin-containing extracts of the *Ch. globosum* MTAV35 and MTAV37 strains were measured in resting sperm cells exposed at 24 °C for two days. Three criteria were assessed: (1) the motility inhibition of resting boar spermatozoa (BSMI_R_); (2) the change in mitochondrial membrane potential (ΔΨ) observed using the potentiometric dye JC-1; and (3) the plasma membrane integrity (SMID_R_) observed using the viability stain calcein-AM + propidium iodide (PI). Chaetoglobosin A and the *Ch. globosum* extracts of the MTAV35 and MTAV37 strains exhibited equally low EC_50_ concentrations (3–4 µg mL^−1^) in the motility inhibition assay and mitochondrial depolarization assay, during which the resting spermatozoa were exposed. By contrast, the EC_50_ concentrations in the SMID_R_ assays, which measured the lethal damages in plasma membrane after incubation for two days, were >50 times higher (Figure 5 and Table 3). Therefore, chaetoglobosin A and the chaetoglobosin-containing extracts also provoked a toxic response after the prolonged exposure, indicating that their sublethal toxicity differs from the lethal toxic responses elicited by alamethicin [41].

The mitochondria were depolarized in sperm cells exposed to sublethal concentrations of chaetoglobosin A (Figure 5) and to the chaetoglobosin-containing extracts (Table 3). This observation aroused the suspicion regarding mitochondrial toxicity. The mitochondrial toxicity of the extracts containing chaetoglobosin was assessed by measuring the accelerated glucose consumption in resting PK-15 cells. Exposure to these chaetoglobosin-containing extracts at concentrations between 50 and 0.5 µg mL^−1^ did not promote the acceleration of glycolysis in the exposed PK-15 cells. By contrast, the positive controls for mitochondrial toxicity—enniatin B, moniliformin and valinomycin at sublethal concentrations of 1.5, 4 and 5 ng mL^−1^, respectively—promoted the acceleration of glycolysis. This result indicates that no impaired mitochondrial functions based on the acceleration of glycolysis in the PK-15 cells exposed to chaetoglobosins [42] were detected. The chaetoglobosin-containing extract thus exhibited a unique sublethal profile which differed from that exhibited by the sublethal mitochondrial toxins valinomycin [43], enniatin B [44] and moniliformin [11] in terms of bioactive responses in the set of tests used.

### 2.5. Resistance of Indoor Chaetomium Strains to Biocides and the Wetting Agent Genapol-X-080 

Eleven *Ch. globosum* isolates obtained from urban indoor environments, one *Ch. globosum* strain obtained from a piggery and two urban indoor *Ch. cochliodes* strains were tested for their resistance to biocides and Genapol -X080 (a wetting agent used indoors). Two mammalian cell lines, PK-15 and MNA, as well as representative strains of the genera *Trichoderma, Aspergillus*, *Paecilomyces* and *Penicillium*, were used as reference strains (*n* = 36). The endpoints measured using the microscopic germ tube test (Figure S3) and based on turbidity and fluorescence emission, resporulation (Figures S4 and S5) and glucose consumption were similar for the tested chemicals, namely borax, Boracol, polyhexamethylene biguanide hydrochloride (PHMB), Genapol-X080, phenoxyethanol, chloramine and triclosan. The SDs among 3–4 independent measurements were between 38% and ≤28.5%. The toxic endpoints for conidial germination and for resporulation for all chemicals were similar, except for Genapol-X080 where resporulation was inhibited at concentrations lower by 100 times relative to the concentrations in the other endpoints. The results are shown in Figures S4–S6 and in Table 4. The EC_50_ values in Table 4 represent a value between EC_0_ and EC_100_ and the median of four independent measurements with five methods. Genapol-X080 differed from the other tested chemicals in that the endpoints for conidial germination measured based on germ tube test, turbidity, fluorescence emission and glucose consumption were >100 times higher than the endpoint for resporulation. The high sensitivity to Genapol-X080 by the *Ch. globosum* HAS5 strain is illustrated in Figure S4; the toxic endpoint of Genapol-X080 expressed as the average EC_50_ values of four measurements was 44 µg mL^−1^ (SD ± 12.5). The reference strain *Trichoderma atroviride* H1/226 exhibited an EC_50_ value of ≥50,000 µg mL^−1^ for Genapol-X080.

The high sensitivity of the *Ch. globosum* strain MTAV35 to Genapol-X080 relative to that of the selected *Aspergillus* reference strains is visualized in Figure S5. The figure shows bright fluorescence emitted by all dilutions in Figure S5A,B (Row 4). No fluorescence was emitted by the dilutions in Figure S5C (Row 4). This result illustrates that the ascospores of the *Ch. globosum* strain MTAV35 were inhibited in Genapol-X080 dilutions stronger by over 1000 times compared with the conidia of *A. westerdijkiae* PP2 and *A. calidoustus* MH4. Moreover, conidial germination was indicated by the increased turbidity in Figure S5D–F, where the three *Aspergilli* were resistant to the highest concentration—50,000 µg mL^−1^—which is the smallest Genapol-X080 dilution in Row 4. 

Figure S5G–I shows no resporulation in Row 4 at Genapol-X080 concentrations higher than 200–400 µg mL^−1^. The microscopic inspection and turbidimetric estimation revealed the conidial germination in all tested dilutions in Row 4, but resporulation was obviously inhibited or delayed by Genapol-X080. Genapol-X080 differed from the other tested chemicals in that the endpoints for conidial germination were >100 times larger than those for resporulation.

The twelve blue/green fluorescent *Ch. globosum*, the blue fluorescent unidentified *Chaetomium*-like strain Ch1/tu and the somatic cell lines were 1000 times more sensitive to Genapol-X080 (EC_50_ ≤ 50 µg mL^−1^) than the two non-fluorescent *Ch. cochliodes* and the yellow fluorescent *Ch. rectangulare* strains (EC_50_ ≥ 50,000 µg mL^−1^). In addition, our results indicate that the *Ch. globosum* strains and the somatic cell lines were 1000 times more sensitive to Genapol-X080 than the reference strains belonging to *Trichoderma, Aspergillus, Paecilomyces* and *Penicillium* (Figures S4, S5 and Table 4). 

All tested fungal strains, including the *Ch. globosum* strains, were more resistant to borax, Boracol, PHMB and chloramine than the somatic cell lines MNA and PK-15. Phenoxyethanol and triclosan exhibited the smallest differences in terms of the sensitivity of fungi and somatic cells towards them. The most resistant to borax and Boracol were the *Ch. globosum* strains and the *Aspergillus* reference strains. The *Aspergillus* strains also exhibited a high resistance to PHMB, whereas the *Trichoderma* strains exhibited a high resistance to triclosan and chloramine. No difference in resistance pattern was observed between the indoor and outdoor *Trichoderma atroviride* strains. The opportunistic human pathogenic *T. longibrachiatum* and *T. citrinoviride* were more sensitive to PHMB and Boracol than the *T. atroviride* strains. 

In summary, the tested blue/green fluorescent *Ch. globosum* strains isolated from different buildings exhibited a uniform mycotoxin profile, a uniform sublethal toxicological profile and a uniform resistance profile towards detergents and biocides for indoor use. The two non-fluorescent *Ch. cochliodes* strains differed in all four characteristics from the tested *Ch. globosum* strains.

## 3. Discussion

This study revealed that fluorescence emission and toxic responses in two screening assays of biomass dispersal are useful in rapid screening procedures for tracking the diversity of toxigenic indoor ascomata-producing *Chaetomium*-like isolates. In addition to the most abundant species *Ch. globosum*, two other species—*Ch. cochliodes* and *Ch. rectangulare*, which have not been previously investigated in buildings in Finland—were found. The blue/green fluorescent (morphotype A in Figure 2) and non-fluorescent (morphotype B in Figure 2) strains exhibiting unbranched ascomatal hair were identified as toxigenic *Ch. globosum* and *Ch. cochliodes*, respectively, which produce chaetoglobosin and chaetomin, as the dominant mycotoxins detected. The blue fluorescent isolates (morphotype C in Figure 2) exhibiting dichotomously branched ascomatal hair and small ascospores were tentatively identified down to the genus level based on their morphology. These strains were isolated from an inlet air filter and possibly came from the outdoor air (Table 1). The yellow fluorescent toxigenic isolates (morphotype D in Figure 1) exhibiting dichotomously branched ascomatal hair and large ascospores were identified as *Ch. rectangulare*, and its toxin production, as well as the biological activity of the produced toxins and the possible correlation of these toxins to piglet mortality [34], will be investigated in a separate paper.

The indoor-isolated *Ch. globosum* and *Ch. cochliodes* strains were shown to produce mycotoxins, including chaetoglobosin, chaetoviridin A and C, chaetomugilin D and chaetomin, on laboratory media and building materials, suggesting that mycotoxin production is not species-specific [2,9,28,45]. In our study, there is a clear difference in mycotoxin production between *Ch. globosum* and *Ch. cochliodes*. For instance, the amount of chaetomin in *Ch. cochliodes* extracts was one-fourth that of chaetoglobosin in the *Ch. globosum* extracts. However, in the ICP assay, the EC_50_ concentrations of the chaetomin-containing extracts were 100 times lower compared with that of the chaetoglobosin-containing extracts. By contrast, their EC_50_ concentrations in the BSMI assay were similar. This result indicates that the chaetomin-containing extracts were more toxic to the cell lines used or that chaetoglobosin was more susceptible to inactivation during the extraction process; for instance, chaetoglobosin could be inactivated by the heat treatment and ethanol extraction needed for ascospore inactivation. For chaetomin, its sperm motility-inhibiting effect may be explained in terms of mitochondrial toxicity. Chaetomin is known to induce cell apoptosis by disrupting the mitochondrial function and by promoting calcium overload [46]. For chaetoglobosin, its sperm motility-inhibiting effect and cytostatic effect could be very likely explained by its glucose transport-inhibiting activity [23,33]. This result indicates that the chaetoglobosin did not target mitochondria. Thus, energy depletion caused by inhibition of sugar transport may be monitored as a sublethal toxicity in boar sperm [32]. The chaetoglobosin-producing *Ch. globosum* strains apparently excrete chaetoglobosin in liquid vesicles (Figure 5 and Table 3).

Although the number of indoor isolates in this study was limited, the results indicate that the chaetoglobosin-producing *Ch. globosum*-like strains are the most common but not the only ascomata-producing fungi in the investigated buildings in Finland. In addition, the most commonly isolated indoor *Chaetomium* species worldwide is *Ch. globosum* [2,3]. To the best of our knowledge, chaetomin-producing indoor *Ch. cochliodes* or *Ch. rectangulare* isolates have not yet been reported in Finland.

*Ch. globosum* was shown to cause changes in the structure and porosity of plywood and concrete, allowing them to penetrate building materials in search for nutrients. Over time, building materials will become more fragile, resulting in construction issues [5,47]. Such a characteristic is possibly the reason fungal growth is difficult to eradicate. The results demonstrate the variability among indoor fungal species and genera in terms of biocide/chemical resistance. All of the tested chaetoglobosin-producing strains but none of the tested *Aspergillus*, *Trichoderma, Paecilomyces* or *Penicillium* strains were sensitive to Genapol-X080. It is tempting to speculate about a correlation between chaetoglobosin production and Genapol-X080 sensitivity. Interestingly, no difference in resistance pattern was observed between the indoor and outdoor strains of *T. atroviride*. Biocide resistance seems to be a species-specific characteristic. The indoor use of biocides and chemicals may influence the proliferation and species diversity of the indoor microbiota.

## 4. Conclusions

The tested blue/green fluorescent *Ch. globosum* strains isolated from eight buildings exhibited a uniform mycotoxin profile that was dominated by chaetoglobosin. The isolates exhibited a uniform sublethal toxicological profile, which is attributed to energy depletion. The isolates displayed a uniform resistance profile towards indoor detergents and biocides, but a profile which differed from that of other species that are sensitive to Genapol-X080. The two non-fluorescent, chaetomin-producing *Ch. cochliodes* and the two yellow fluorescent *Ch. rectangulare* strains differed from the tested *Ch. globosum* strain in all characteristics. This diversity based on fluorescence and biocide/Genapol-X080 resistance was confirmed by toxicity and metabolite profiling and by the *tef1α* sequence-based identification. Thus, fluorescence, biocide/Genapol-X080 resistance, and toxicity screening are potentially useful in the preliminary diversity tracking of mycotoxinogenic isolates belonging to the *Ch. globosum* complex. 

## 5. Materials and Methods

### 5.1. Experimental Design

New methods for tracking the diversity of toxigenic ascomata-producing colonies cultivated from indoor dusts were developed. These methods were employed to detect and characterize the indoor *Chaetomium* strains. Settled dust and inlet air filters were collected from urban and rural buildings associated with severe health problems in Finland. Biomass suspensions of mold colonies from the primary cultivation plates were screened using two complementary rapid toxicity tests. The colonies found to be toxic in the two assays were examined under a light microscope for ascomata formation. The biomass suspensions prepared from these colonies were inspected for fluorescence emission.

The toxigenic ascomata-producing colonies from the primary isolation plates were streaked into pure, single-spored cultures and classified into four morphotypes denoted as A–D based on the morphology of their ascomata and on their fluorescence emission. Representatives of morphotypes A–C from different buildings were identified down to the species level. The strains exhibiting morphotypes A and B and belonging to the genus *Chaetomium* were investigated for mycotoxin production. The mycotoxins in the extracted biomass were analyzed and identified with HPLC-MS. The biological effects and targets of the toxins were elucidated in a set of four bioassays and then compared with selected pure mycotoxins from selected reference indoor fungi. The sensitivity of the *Chaetomium* strains to biocides and to the wetting agent Genapol-080 was compared with that of selected representatives of indoor and outdoor fungal species and genera. 

### 5.2. Cultivation of Mold Colonies

The methods used for cultivating the mold colonies on malt extract agar (15 g malt extract from Sharlab, Spain, and 12 g of agar from Amresco, Solon OH, USA, in 500 mL of H_2_O) were described previously [17,18]. After three weeks of incubation, the colonies on the primary isolation plates (not yet single spored) were numbered and screened for toxicity [17,18].

### 5.3. Rapid Screening Tests Using Ex Vivo and In Vitro Assays

The toxicity tests measured: (a) the toxins affecting the cellular energy metabolism, the mitochondria and ion homeostasis based on the inhibition of the motility of boar spermatozoa (BSMI) [13,15,16,48]; and (b) the toxins affecting macromolecular synthesis and cytostatic activity based on the inhibition of the proliferation of the somatic cell line PK-15 (ICP) [49]. The screening tests that were applied directly to the primary sampling plates and designed for mycotoxinogenic indoor molds were described previously [34]. In this study, the procedure of the test was briefly as follows: 10–20 mg of colony biomass were suspended in 200 µL of ethanol and heated in a water bath to 55–60 °C for 10 min. In the ex vivo test, the rapid BSMI assay red after 30 min of exposure, 200 µL of extended boar semen were exposed to 5 µL of biomass suspension. In the slower version of the test, when 1 and 3 days of exposure were used, 2 mL of extended boar semen were exposed to 10 µL of biomass suspension. The sperm cells were exposed at 24 °C. A colony was considered very toxic in the BSMI assay when ≤2.5 vol% of its biomass suspension inhibited the boar sperm motility after 30 min to 1 day exposure and slightly toxic if the motility inhibition occurred after 3 days of exposure. A colony was considered toxic in the in vitro ICP assay when ≤5 vol% inhibition of cell proliferation (ICP) assays the proliferation of the porcine kidney (PK-15) cells after 2 days of exposure, respectively.

### 5.4. Toxicity Assays for Ethanol-Soluble Dry Substances Extracted from a Plate-Grown Fungal Biomass and Pure Mycotoxins

The plate-grown biomass of the fungal pure cultures (100–300 mg) was extracted with ethanol as described earlier [31]. The toxicity assays, namely BSMI assay after exposure for 30 min (rapid inhibition) and for one day (slow inhibition), SMID assay and ICP assay, were all described previously [15,16]. The toxicity assays involving the ethanol extracts obtained from pure fungal cultures were performed using porcine cells (sperms and somatic cell lines) as indicators according to methods described before [13,18,41,50]. The calculation of EC_50_—the half maximal effective concentration for the ethanol-dry substances and pure mycotoxins in the BSMI, SMID and ICP assays—were described previously [15,18].

The procedure for the BSMI_R_ assay measuring sublethal toxicity as motility inhibition, i.e., inability to respond to induction of motility in resting sperm cells is described below: The ethanol solutions (0.5–10 μL) were dispensed in 2000 μL of extended boar semen (Figen Ltd., Tuomikylä, Finland, density of 27 × 10^6^ sperms mL^−1^) incubated at 24 °C, and motility of the sperms was inspected in the phase-contrast microscope (400× magnification) with a heated stage, (Olympus CKX41, Tokyo, Japan; magnification 400×) and an image recording software (Cellsense^®^ standard version 11.0.06, Olympus Soft Imaging Solutions GmbH, Münster, Germany, 2012) as described previously [34]. Briefly, the EC_50_ concentration for motility inhibition was concluded as the toxin concentration closest to that provoking a > 50% decrease in the number of sperm cells exhibiting rapid tail beating, compared with the sperm cells in the solvent control. The EC_50_ was calculated from the equation of the straight line between EC_50-40_ and EC_80-90_: Y = −ΔY/ΔX × X + C where Y is the motility closest to 50% of the motility of the solvent control, X is the EC_50_ concentration and C is a constant between 100% and 60%. All tests were run in triplicates and differences between replicate tests were within one dilution step (2-fold). The sperm assays were calibrated with triclosan and valinomycin. 

The boar sperm motility inhibition assay with motile spermatozoa (BSMI_M_) measuring inability to maintain induced motility was performed as follows: Motile sperm cells were exposed to dilutions of the biomass extracts and exudates at 37 °C for 20 min. Aliquots of 200 μL of extended boar semen were exposed to 0.5, 1 and 2 μL of ethanol-soluble compounds from ten-fold dilutions of biomass extracts or exudates. Estimation of the ratio of motile spermatozoa compared to the control and calculation of EC_50_ was done as in the BSMIR assay described above.

Lethal toxicity was measured as sperm membrane integrity disruption assay with motile spermatozoa (SMID_M_), was assessed by staining with PI as described [18] with modifications. Aliquots of 50 μL PBS were pipetted into a microtiter plate. Ethanol-soluble compounds from biomasses (50 μL) of fungal strains were serially diluted to 10^9^, and extended boar cell aliquots (150 μL) were added to the wells. The possible autofluorescence of the toxins was excluded. PBS was used as a blank reagent. Three parallel dilutions were performed for each sample. Frozen–thawed semen only exposed to ethanol was used as a positive control (100% mortality) representing the maximal fluorescence emitted by the cells permeable to PI. Sperm cells only exposed to ethanol were used as a negative control (viable cells). The microtiter plate was pre-incubated for 2 h at 37 °C on an orbital shaker (Innova 5000 New Brunswick Scientific, Enfield, CT, USA) at 160 rpm. A volume of 100 μL PI solution (10 μg mL^−1^) was added to each well of the microtiter plate. The plate was incubated for 15 min at 37 °C in the dark. Fluorescence was measured with a microplate reader (Fluoroskan Ascent, Thermo Scientific, Vantaa, Finland) at excitation and emission wavelengths of 544 and 590 nm, respectively.

The test for mitochondrial damage as indicated by accelerated glucose consumption and extracellular acidosis was described earlier [24,51,52]. The PK-15 cells were grown in 8-well flat-bottom chamber glass slides, seeded to a density of 4 × 10^4^ cells mL^−1^, in the respective medium for 48 h. The twofold serially diluted test substances or the vehicle only was dispensed into the wells. The glucose content of the wells was measured by using a glucose meter (Precision Xceed; Abbott Diabetes Care Ltd., Berkshire, United Kingdom). In wells that received nothing or the vehicle only, the glucose concentration decreased from an initial concentration (0 h) of 12 to 6 mM (24 h), but the pH remained as pH of 7.2. A glucose concentration after 24 h of ≤3 mM indicated excessive glucose consumption. Prior to the measurement of the pH, the chamber slides were transferred into ambient air for 1 h to allow CO_2_ to evaporate. A pH drop of 0.5 units in the toxin-exposed well compared to the vehicle only was considered proof of acidosis. Mitochondrial depolarization (ΔΨm assay) was read by a fluorescence microscope after 26 h of exposure to the toxicants from the monolayers double stained with the membrane potential-responsive fluorogenic dye JC-1 and with propidium iodide to assess the relaxing of the plasma membrane permeability barrier, similar to what was reported previously for sperm cells (27). 

### 5.5. Fluorescence Microscopy of Resting Spermatozoa Exposed in the SMID_R_ and the ΔΨm Assays

The boar sperm cells exposed for 2 days to commercial chaetoglobosin A and the crude extract of MTAV35 and MTAV37 were double-stained with the viability stains PI + calcein–AM, the SMID_R_ assay, as described earlier [53]. The staining protocol was as follows: 200 μL of extended boar semen containing 27 × 10^6^ sperm cells mL^−1^ was mixed with 200 μL PBS containing 3 μg mL^−1^ calcein-AM and 10 μg mL^−1^ PI. The ΔΨm assay monitored the mitochondrial membrane potential changes (ΔΨm) by staining with the lipophilic potentiometric dye JC-1 as described by Mikkola et al. (2015). For the staining with JC-1 (35 µg mL^−1^), 200 µL of the sperm cells were shaken and incubated at 37 °C for 5 min. After staining, the cells exposed in the SMID_R_ and the ΔΨm assays were inspected with fluorescence microscope using 400× magnification (Nikon Eclipse E600, Nikon Corporation, Tokyo Japan) with filters BP 450–490 nm/LP 520. The calcein–AM-stained cells with intact plasma membranes produced green fluorescence, whereas the PI-dyed cells with damaged plasma membranes produced red fluorescence. In JC-1 stained sperm cells, mitochondria with high membrane potential emitted yellow fluorescence, whereas depolarized mitochondria fluoresced green.

The EC_50_ concentration in these microscopic assays was defined as the lowest concentration where the ratio of cells similar to those in the solvent control was less than 50%. This EC_50_ fitted between EC_90_ and EC_10_ observed in the microscope calculating ca. 100–120 sperm cells from three microscopic fields. The maximal difference between four parallel tests in each of the two methods was one dilution step. The assays were calibrated with triclosan.

### 5.6. Identification of the Fungal Strains

The isolates were initially identified down to the genus level based on colony morphology on MEA, on conidiophore morphology as seen under a light microscope, and on conidial size [54].

The *Ch. globosum* MH52, HAS5, 2b/26, C22/LM, RUK10 and MO9 strains; the *Ch. cochliodes* OT7 and OT7b strains; and the *Ch. rectangulare* MO13 and MO15 strains were identified in the present study based on the amplification of a fragment of the translation elongation factor 1 alpha (*tef1*α) gene as described earlier [55], (GenBank Accession numbers: MT498101-MT498110).

The reference strains *A. westerdijkiae* PP2 and PP3, *A. versicolor* SL/3 and GAS/226, *Ch. globosum* MTAV35 and *Paecilomyces variotii* Paec 1/kop and Paec 2/kop were identified at DSMZ (Deutsche Sammlung von Mikroorganismen und Zellkulturen) between 2004 and 2008. The *T. atroviride* strains Ke14 [6], KIV10, Tri335, 14/AM and H1/226 as well as the strains *T. citrinoviride* SJ40 [25] and *T. longibrachiatum* HAMBI 3643 (=SzMC Thg) [56] were identified previously based on internal transcribed spacer (ITS) and/or *tef1*α sequence analysis. The identification of *A. calidoustus* MH4 based on ITS was described earlier [34]. 

The outdoor *T. atroviride* strains were obtained from the Szeged Microbiology Collection (www.szmc.hu, University of Szeged, Hungary).

### 5.7. HPLC-MS Analysis and Identification of Mycotoxins in the Fungal Extracts

The biomass (400 ± 50 mg) of the selected *Ch. globosum* and *Ch. cochliodes* strains was harvested from a malt extract agar (MEA) plate incubated at 24 °C for 14 days. The fungal toxins were extracted with ethanol from the collected biomass, and the ethanolic extract was analyzed by high-performance liquid chromatography–mass spectrometry (HPLC-MS). The HPLC-electrospray ionization ion trap mass spectrometry analysis (ESI-IT-MS) was performed using an MSD-Trap-XCT plus ion trap mass spectrometer equipped with an Agilent ESI source and Agilent 1100 series LC (Agilent Technologies, Wilmington, Del., USA) in positive mode with the mass range of *m/z* 50–2000. ESI source parameters used for analysis were: nebulizer gas pressure, 35 psi; drying gas flow rate, 8 L min^−1^; drying gas temperature, 350 °C; and a capillary voltage of 5000 V. The column used was a SunFire C18, 2.1 × 50 mm, 2·5 μm (Waters, Milford, MA, USA). Separation of compounds from the ethanol extract of biomasses of *Ch. globosum* and *Ch. cochliodes* strains was done using an isocratic method of solution A: H_2_O with 0·1% (v/v) formic acid and B: methanol in a ratio of 40/60 (*v*/*v*) for 15 min and gradient of 100% B for 50 min at a flow rate of 0.2 mL min^−1^. Identification and quantification of compounds was done by HPLC-UV-MS and MS/MS analysis. 

### 5.8. Methods for Testing the Toxicity of Biocides and Genapol-X080 towards the Fungi

The toxicity of biocides and chemicals towards the indoor *Chaetomium*-like isolates and the reference indoor strains of the genera *Aspergillus, Paecilomyces, Penicillium* and *Trichoderma* was assessed in parallel with the six parameters presented in the Supplementary Materials: (1) germ tube assay (Figure S3); (2) estimation of turbidity (Figure S4); (3) visual emission of fluorescence (excitation at 360 nm); (4) visual resporulation of conidia; (5) inhibition of glucose consumption [51]; and (6) resazurin reduction. All assays were performed in malt extract broth (12 g L^−1^ malt extract, 7.2 µmol mL^−1^ glucose, pH 5.5; Scharlab, Barcelona, Spain) in 96-well microtiter plates as described for the ICP assay.

The assays measuring inhibition of germination of conidia were done with spore suspensions of 1–2 × 10^6^ fungal spores mL−^1^. The tests were performed in malt extract broth (Oxoid, Hampshire United Kingdom) 12 g in 1 L, pH 5.5, glucose 7.1 mmol L^−1^, and performed on microtiter plates as described by Bencsik et al. [3] for the ICP assay. The germ tube germination is visualized in Figure S3, and the tests estimating turbidity and emission of fluorescence provoked by the germinated and metabolically active conidia and *C. globosum* ascospores are visualized in Figures S4 and S5. Examples of the tests estimating fluorescence emission, turbidity and re-sporulation of conidia and ascospores are compared in Figure S5. The difference of the endpoints measured with the different assays was between 38% and <28.5%. The resazurin reduction assays (Figure S6) were performed as described for the ICP assay, except that the microtiter plates were visually inspected for the change of the blue/violet color of resazurin (indicating inhibition of germination of the exposed conidia) to the colorless dihydroresorufin (indicating metabolic activity and germination of the exposed conidia).

The formation of germ tubes was inspected by a phase-contrast microscope (Olympus CKX41, Tokyo, Japan; magnification 400×) and an image recording software (Cellsense^®^ standard version 11.0.06, Olympus Soft Imaging Solutions GmbH, Münster, Germany, 2012) after 1 and 2 days of incubation at 28 °C. The EC_50_ corresponded to the inhibition of 50% (estimated as the concentration between EC_0_ and EC_100_) of the conidia compared to the ethanol control (the growth medium containing 1% of ethanol). The tests were run in triplicate and calibrated with triclosan (CAS: 3380-34-5; Sigma Chemical Co., St. Louis, MO, USA) giving a mean SD of ±<20%.

### 5.9. Reagents and Supplies

The continuous cell line PK-15 (porcine kidney cells) and a malignant cell line (MNA cell line) provided by EVIRA (Helsinki, Finland) were used in the ICP assay. 5,5′,6,6′-tetrachloro-1,1′,3,3′-tetraethyl-benzimidazolylcarbocyanine iodide (JC-1, 3520-43-2), PI (25535-16-4) and calcein-AM (148504-34-1) were obtained from Invitrogen (Carlsbad, CA, USA). Alamethicin A4665 (*T. arundinaceum*) (CAS 27061-78-5), a mixture of alamethicin F50 peptaibols (MW 1962, 1976, 1976 and 1990), chaetoglobosin A (CAS 50335-03-0), sterigmatocystin (CAS 10048-13-5) and ochratoxin A (CAS 303-47-9) were obtained from Sigma-Aldrich (St. Louis, MO, USA). Malt extract and tryptic soy agar were obtained from Scharlab (Barcelona, Spain). The other chemicals were of analytical grade and were purchased from local suppliers. The biocides and the wetting agent Genapol-080 were purchased from Sigma-Aldrich Finland. The fungicide Boracol 10Rh was obtained from a local supplier. The selected biocides were Borax (600 µg mL^−1^; sodium tetraborate, CAS: 1330-43-4), Boracol 10Rh containing disodium octaborate tetrahydrate (CAS: 12280-03-04) and didecyldimethylammonium chloride [CAS: 7173-51-5]), PHMB (CAS: 27083-27-8), phenoxyethanol (CAS: 122-99-6), chloramine, triclosan (5-Chloro-2-(2,4-dichlorophenoxy)phenol, CAS: 3380-34-5) and the wetting agent Genapol X-080 (CAS: 9043-30-5). Genapol X-080 and the biocides were purchased from Sigma-Aldrich, except for Boracol 10Rh, which was purchased from a local supplier.

## Figures and Tables

**Table 1:** Characterization of 42 ascomata-producing indoor isolates obtained from eight buildings associated with indoor air-related health problems in Finland. The strains were screened to be toxic in the boar sperm motility inhibition and inhibition of cell proliferation bioassays.

Strain Code	Origin	Sampling Site	Fluorescence/Morphotype		Species
MTAV35 *, MTAV37	Settled dust	University, Oulu	Blue-green	A	*Chaetomium globosum*
ABCD	Settled dust	Apartment Helsinki	Blue-green	A	*Ch. globosum*
MH5 †, M13, M14, M15, M16, L17, L18, MTA1, MTA2, MH52 †, MH10. MH10b HAS5, MH12	Settled dust	University Espoo	Blue-green	A	*Ch. globosum*
RUK10 †, R7, R8, R9, R11, R20, R21	Settled dust	Apartment A Vantaa	Blue-green	A	*Ch. globosum*
2b/26 † 2c/MT	Settled dust	Apartment B Vantaa	Blue-green	A	*Ch. globosum*
3b/AP	Exhaust air filter	University Espoo	Blue-green	A	*Ch. globosum*
C22/LM †, C23/LM, C21/LM C14/LM, C4/LM	Exhaust air filter	School B Vantaa	Blue-green	A	*Ch. globosum*
MO9 †, MO10, MO11, MO16	Settled dust	Piggery ‡, Orimattila	Blue-green	A	*Ch. globosum*
OT7 †, OT7b †	Settled dust	Office Helsinki	None	B	*Ch. cochliodes*
Ch1/tu, Ch2/tu, Ch3/tu, Ch4/tu	Inlet air filter	University Espoo	Blue	C	Unidentified *Chaetomium*-like strains
MO15 †, MO13 †, MO12	Settled dust	Piggery ‡, Orimattila	Yellow-green	D	*Ch. rectangulare*

* Identified by Deutsche Sammlung von Mikroorganismen und Zellkulturen; † identified by *tef1α* gene sequencing; ‡ described in [34].

**Table 2:** Toxic responses of ethanol-soluble compounds in the *Chaetomium globosum* and *Ch. cochliodes* extracts as determined by the boar sperm bioassays BSMI_M_ and SMID_M_ and by the cytostatic ICP assay involving porcine kidney cells (PK-15) and murine neuroblastoma cells (MNA). All target cells were exposed at 37 °C.

Strain	Code	EC_50_ (µg dry wt mL^−1^)				Identified Metabolite	Estimated Concentration (mg mL^−1^)
		BSMI_M_	SMID_M_	ICP			
		Boar Sperm		PK-15	MNA		
		20 min	2 h	2 days	2 days		
Biomasses							
Group I blue green fluorescent							
*Ch. globosum*	MTAV35	5	450	40	20	chaetoglobosin	3.4
						chaetoviridin A	0.02
						chaetoviridin C	0.2
*Ch. globosum*	MTAV37	10	350	30	15	No data	
*Ch. globosum*	HAS5	5	310	50	25	chaetoglobosin	3.9
						chaetoviridin A	0.5
						chaetoviridin C	0.2
*Ch. globosum*	RUK10	5	300	20	10	chaetoglobosin	4.2
						chaetoviridin A	0.04
						chaetoviridin C	0.05
*Ch. globosum*	ABCD	5	450	30	15	chaetoglobosin	4.24
						chaetoviridin A	0.3
						chaetoviridin C	0.05
Group II Non-fluorescent							
*Ch. cochliodes*	OT7	10	480	0.5	0.5	chaetomin	1.3
						chaetoviridin A	0.13
						chaetomugilin D	0.02
	OT7b	10	480	0.8	0.8	chaetomin	1.2
						chaetoviridin A	0.3
						chaetomugilin D	0.2
Exudate	2c/MT					Chaetoglobosin ^1^	
Reference mycotoxin						Biological activity	
Alamethicin (*Trichoderma arundinaceum*)		5	1	8	8	Lethal toxin, K^+^ and Na^+^ ion channel former [41]	
Chaetoglobosin A (*Ch. globosum*)		1	12	3	1	Sublethal toxin, inhibitor of glucose transport [42]	
Citrinin (*Penicillium citrinum*)		>100	50	10	10	Cytostatic toxin, nephrotoxic [41]	
Sterigmatocystin (*Aspergillus* spp.)		>20	>20	0.5		Cytostatic toxin, inhibitor of protein synthesis [42]	
Valinomycin (*Streptomyces griseus*)		0.0001	70	14		Sublethal toxin, mitochondrial toxin, potassium carrier ionophore [43]	

^1^ Chaetoglobosin detected in the exudate collected from the malt extract agar (MEA) plate [6].

**Table 3:** Effective concentrations (EC_50_ µg mL^−1^) for lethal and sublethal toxicity recorded in resting porcine spermatozoa and kidney cells (PK-15) exposed to ethanol extracts obtained from the *Chaetomium globosum* strains and selected reference mycotoxins.

Strain Code	EC_50_ µg mL^−1^							
	Boar Sperm						PK-15	
	Lethal Toxicity		Sublethal Toxicity					
	SMID_R_		BSMI_R_		Depletion of ΔΨ_m_		Acceleration of glycolysis	Acidosis
	2 days	4 days	2 days	4 days	2 days	4 days	1 day	2 days
MTAV35	>20	>20	3	3	6	3	None	None
MTAV37	>20	>20	4	4	4	4	None	None
Reference toxins								
Lethal toxin forming ion channels in the plasma membrane								
Alamethicin	0.2		0.2		0.2		None	None
Sublethal toxin inhibiting glucose transport								
Chaeto-globosin A	>20		1		2		None	None
Sublethal mitochondrial toxins								
Enniatin B	>50		5		5		5 (50) *	5
Moniliformin	ND		2		None		4 (40) *	4
Valinomycin	5		0.00005		0.00005		0.005 (5) *	0.005

* Number in parenthesis is the lethal concentration in resting PK-15 cells.

**Table 4:** Resistance to biocides and to the wetting agent Genapol-X-080 of the *Chaetomium*-like strains and the selected indoor and outdoor fungal strains and mammalian cell lines. Resistance was measured based on conidial and ascospore germination, which were evaluated in terms of the formation of germ tubes as observed under a light microscope, increased turbidity, fluorescence emission and resporulation.

Strain Code	EC_50_ (µg mL^−1^)						
	Borax	Boracol	PHMB	Genapol-X080	Phenoxy-ethanol	Chloramine	Triclosan
Indoor *Ch. globosum* strains							
MTAV35	5000	100	4	<50	700	1200	2
MTAV37	5000	100	4	<50	700	1200	4
HAS5	>5000	100	8	50	1500	2500	2
2b/26,	>5000	100	8	50	1500	2500	4
MH 52	5000	50	4	<50	1500	1200	2
RUK10	5000	100	4	<50	1500	2500	2
ABCD	5000	100	4	<50	1500	1200	4
MO9	>5000	100	4	<50	700	2500	2
2c/MT	>5000	100	8	50	1500	2500	4
C22/LM	>5000	100	8	50	1500	2500	4
3b/AP	>5000	100	8	50	1500	2500	4
Indoor *Ch. cochliodes* strains							
OT7	750	50	8	>5000	3000	ND	4
OT7b	750	50	8	>5000	3000	ND	4
Indoor *Ch. rectangulare* strains							
M015	5000	100	4	50,000	1500	1200	2
Mo13	>5000	100	4	50,000	1500	1200	2
Reference strains							
	Outdoor *Chaetomium-*like strains						
CH1/tu	1200	100	4	<50	1500	1200	2
	Strains that could grow at 37 °C						
	Indoor strains of *Aspergillus* section *Nigri*						
Asp21	≥5000	1600	30	>50,000	3000	1200	30
Asp33b	≥5000	1600	30	>50,000	3000	1200	16
Asp 32	≥5000	1600	60	>50,000	3000	1200	16
	Indoor strains of *Aspergillus* section *Flavi*						
7D	2500	200	120	>50,000	1500	600	8
1/37	5000	1600	120	>50,000	1500	600	8
Indoor *Aspergillus calidoustus* strain							
MH4	5000	200	8	>5000	3200	ND	16
Indoor *Paecilomyces variotii* strains							
Pac1/kop	≥5000	800	30	>50,000	3000	1200	4
Pac2/kop	≥5000	400	30	>50,000	3000	1200	4
	Indoor *Paecilomyces* sp. strains						
Pec1/skk	≥5000	800	16	>50,000	3000	1200	4
Pec1/his	≥5000	400	30	>50,000	3000	1200	30
		Indoor *Trichoderma longibrachiatum* strain					
THG	1200	<50	1	>50,000	1500	1200	16
	Indoor *Trichoderma citrinoviride* strain						
SJ40	1200	<50	2	>50,000	1500	1200	8
	Strains not able to grow at 37 °C						
	*Aspergillus versicolor* strains						
SL/3	5000	100	60	>50,000	800	600	16
Gas/226	5000	100	60	>50,000	400	1200	16
	*Aspergillus westerdijkiae* strains						
PP2	5000	100	60	>50,000	1500	600	16
PP3	5000	100	60	>50,000	1500	600	16
AW/KL	2500	100	60	>50,000	1500	600	16
Indoor *Trichoderma atroviride* strains							
H1/226	1200	100	2	>50,000	1500	1200	16
Ke14	1200	100	4	>50,000	1500	1200	16
KIV10	2500	100	4	>50,000	3000	2400	30
Tri335	2500	100	4	>50,000	3000	1200	30
14/AM	1200	100	4	>50,000	3000	1200	30
Tri7A/SKK	1200	100	8	>50,000	1500	2500	16
Outdoor *T. atroviride* strains							
SZMC 12541	1200	100	2	>50,000	1500	2500	16
SZMC 12474	2500	100	4	>50,000	3000	2500	8
SZMC 207080	1200	100	8	>50,000	3000	2500	8
SZMC 1723	1200	100	8	>50,000	1500	2500	16
SZMC 12516	2500	200	30	>50,000	1500	2500	16
SZMC 12323	1200	100	8	>50,000	1500	2500	30
	Indoor *Penicillium* sp. strains unable to grow at 37 °C						
35/skk	5000	<50	30	>50,000	350	600	ND
26/skk	>5000	<50	30	>50,000	3000	600	30
5/skk	2500	100	8	>50,000	1500	600	2
HJ2	2500	100	1	>50,000	800	1200	8
20b/skk	>5000	<50	30	>50,000	1500	600	2
Vaip/skk	>5000	<50	8	>50,000	800	600	4
Mammalian cell lines (ICT assay)							
MNA	150	<50	4	25	400	80	4–8
PK-15	600	<50	15	25	1500	150	8–15

## Supplementary Materials

The following are available online at https://www.mdpi.com/2072-6651/12/7/443/s1, Figure S1: The MS and MS/MS spectra of the compounds of the *Ch. globosum* RUK10 and *Ch. cochliodes* OT7; Figure S2: UV-spectra of the compounds of the *Ch. globosum* strains MTAV35, HAS5, RUK10 and ABCD and *Ch. cochliodes* strains OT7 and OT7b; Figure S3: Germ tube test performed with *Aspergillus westerdijkiae* strain PP2 exposed for 48 h at 28 °C to Triclosan; Figure S4: Toxicity of selected chemicals visible as lack of turbidity of two-fold dilutions from left to right after 10 d of incubation; Figure S5: Resistance to biocides and Genapol visible as fluorescence emission (upper row), turbidimetry (middle row) and resporulation (bottom row); Figure S6: Resistance of biocides and Genapol to *Trichoderma atroviride* strains estimated as resazurin reduction (Panels A and C) and resporulation (B and D).
