# Supplementary material for: Detection of Chaetomium globosum, Ch. cochliodes and Ch. rectangulare during the Diversity Tracking of Mycotoxin-Producing Chaetomium-like Isolates Obtained in Buildings in Finland
Source: Toxins (Basel). 2020 Jul 8;12(7):443. doi: 10.3390/toxins12070443 (PMC7405012; doi:10.3390/toxins12070443)
Supplement: Supplementary file 1 [file toxins-12-00443-s001.pdf]

Supplementary Materials: Detection of *Chaetomium globosum*, *Ch. cochliodes* and *Ch. rectangulare* during the Diversity Tracking of Mycotoxin-Producing *Chaetomium*-like Isolates Obtained in Buildings in Finland

Johanna M. Salo, Orsolya Kedves, Raimo Mikkola, László Kredics, Maria A. Andersson, Jarek Kurnitski and Heidi Salonen

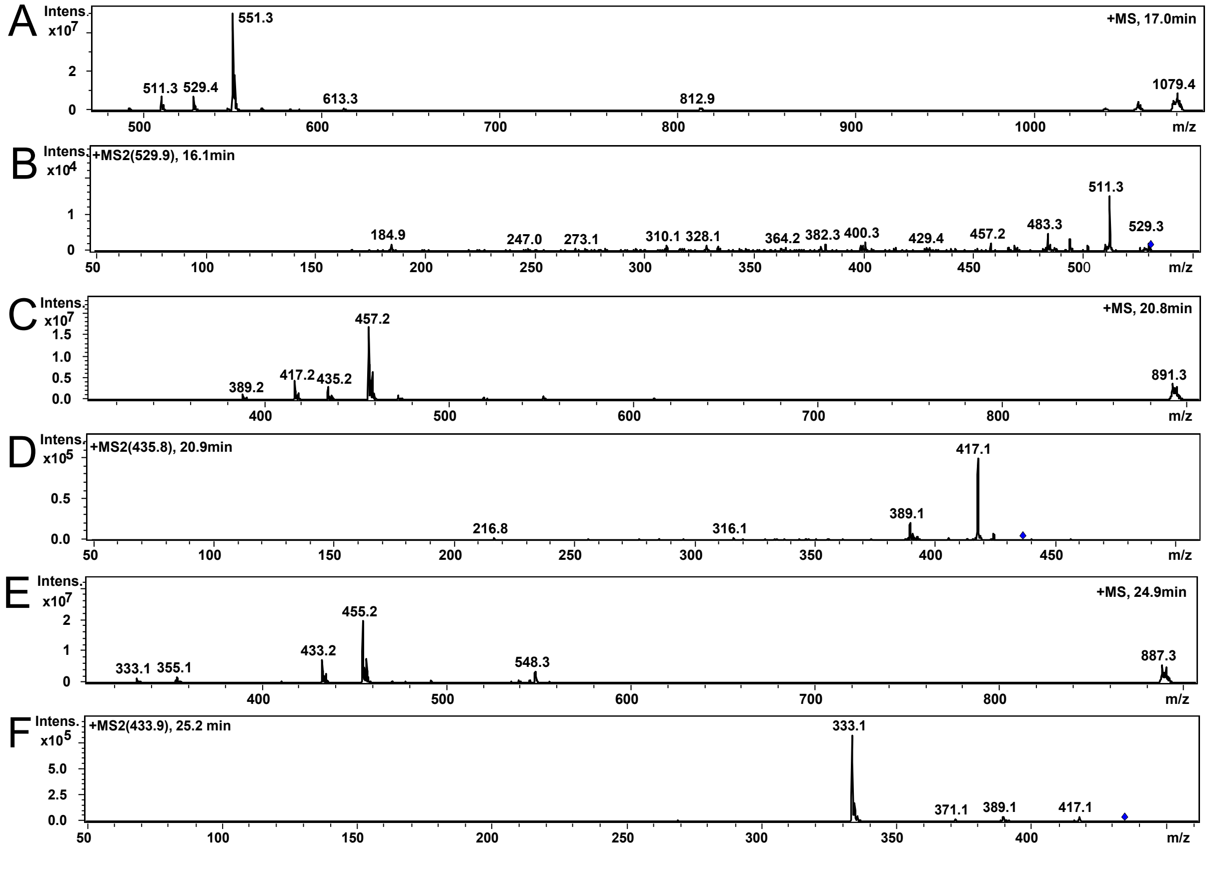

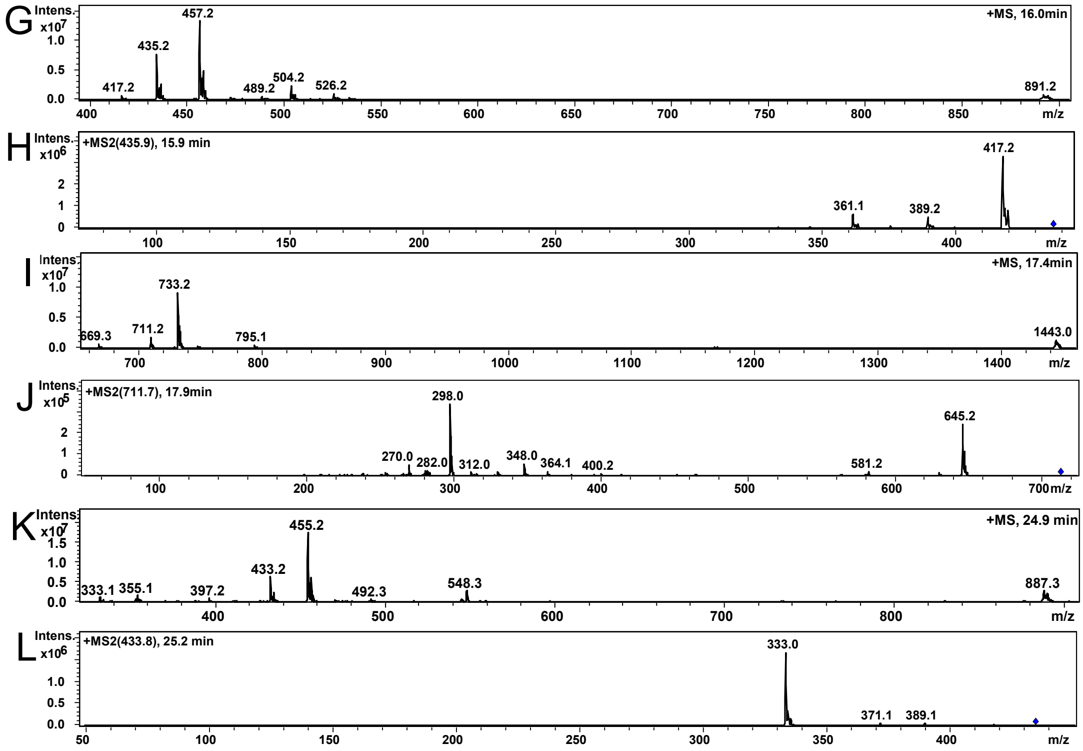

Figure S1. The MS and MS/MS spectra of the compounds of the *Ch. globosum* RUK10 and *Ch. cochliodes* OT7. (**A**) MS spectrum of chaetoglobosin (compound **1**) of the *Ch. globosum* RUK10. (**B**) MS/MS spectrum of chaetoglobosin precursor ion *m*/*z* at 529.9. (**C**) The MS spectrum of chaetoviridin C (compound **2**) from *Ch. globosum* RUK10. (**D**) MS/MS spectrum of chaetoviridin C precursor ion *m*/*z* at 435.8. (**E**) The MS spectrum of chaetoviridin A (compound **3**) of *Ch. globosum* RUK10. (**F**) The MS/MS spectrum of chaetoviridin A precursor ion *m*/*z* at 433.9. (**G**) The MS spectrum of chaetomugilin D (compound **4**) of *Ch. cochliodes* OT7. (**H**) The MS/MS spectrum of chaetomugilin D precursor ion *m*/*z* at 435.9. (**I**) The MS spectrum of chaetomin (compound 5) of the *Ch. cochliodes* OT7. (**J**) The MS/MS spectrum of chaetomin precursor ion *m*/*z* at 711. (**K**) The MS spectrum of chaetoviridin A (compound **3**) of *Ch. cochliodes* OT7. (**L**) The MS/MS spectrum of chaetoviridin A precursor ion *m*/*z* at 433.8. Similar MS and MS/MS spectra of compounds (**1**–**3**) was obtained from *Ch. globosum* strains MTAV35, HAS5 and ABCD as shown in panels A–F and compounds (**4**, **5** and **3**) *Ch. cochliodes* OT7b as shown in panels G-L.

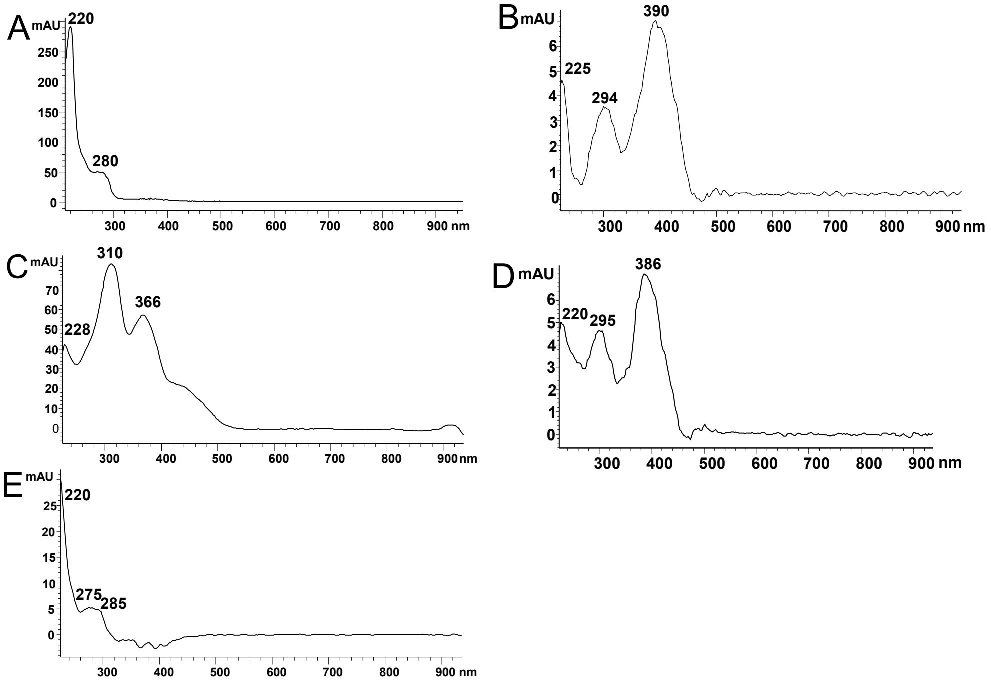

Figure S2. UV - spectra of the compounds of the *Ch. globosum* strains MTAV35, HAS5, RUK10 and ABCD and *Ch. cochliodes* strains OT7 and OT7b. (**A**) Chaetoglobosin (compound **1**), (**B**) Chaetoviridin C (compound **2**), (**C**), Chaetoviridin A (compound **3**) (**D**) Chaetomugilin D (compound **4**) (**E**) Chaetomin (compound **5**).

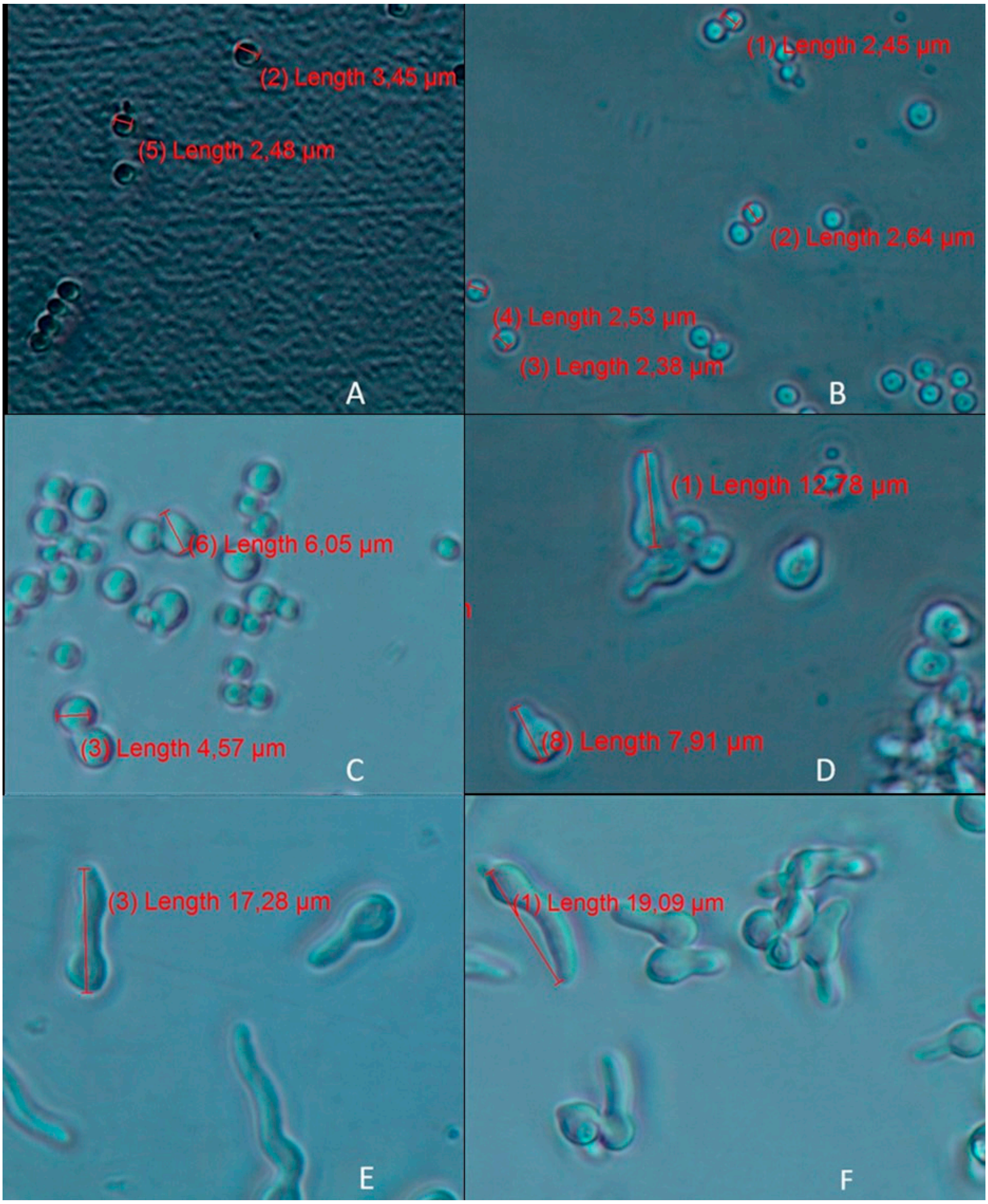

Figure S3. Germ tube test performed with *Aspergillus westerdijkiae* strain PP2 exposed for 48 h at 28 °C to Triclosan. The concentrations of the exposure were as follows: 120 µg mL^{-1} (**A**), 30 µg mL^{-1}(**B**), 15 µg mL^{-1}(**C**), 8 µg mL^{-1}(**D**), 2 µg mL^{-1}(**E**), and the negative control (**F**).

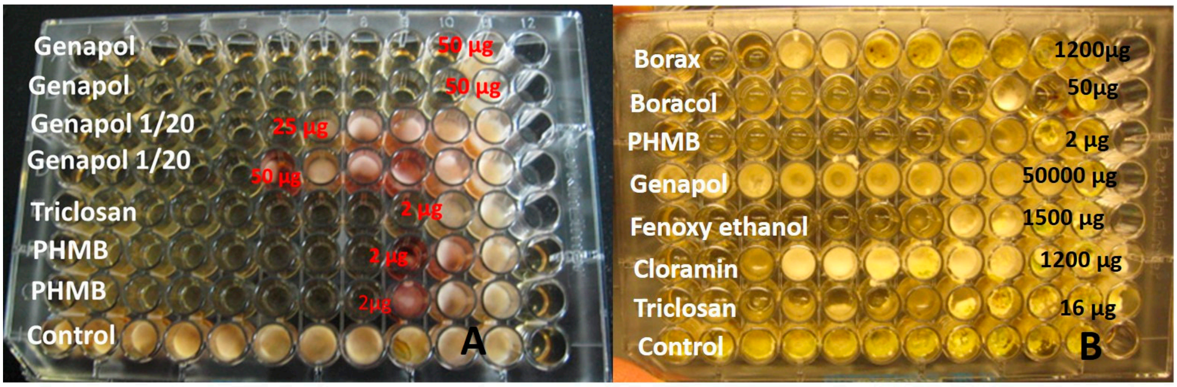

Figure S4. Toxicity of selected chemicals visible as lack of turbidity of two-fold dilutions from left to right after 10 d of incubation. The toxicity, i.e. inhibition of outgrowth of conidia and ascospores is visible as transparency in wells without turbidity. Toxicity endpoints expressed as EC_{50-100} µg mL^{-1}concentrations (values in µg in red and black font) are determined from the concentrations in the last dilution rendering a transparent well. Panel A shows the sensitivity pattern of *Ch. globosum* strain MH5, Panel B shows the sensitivity of *Trichoderma atroviride* H1/226.

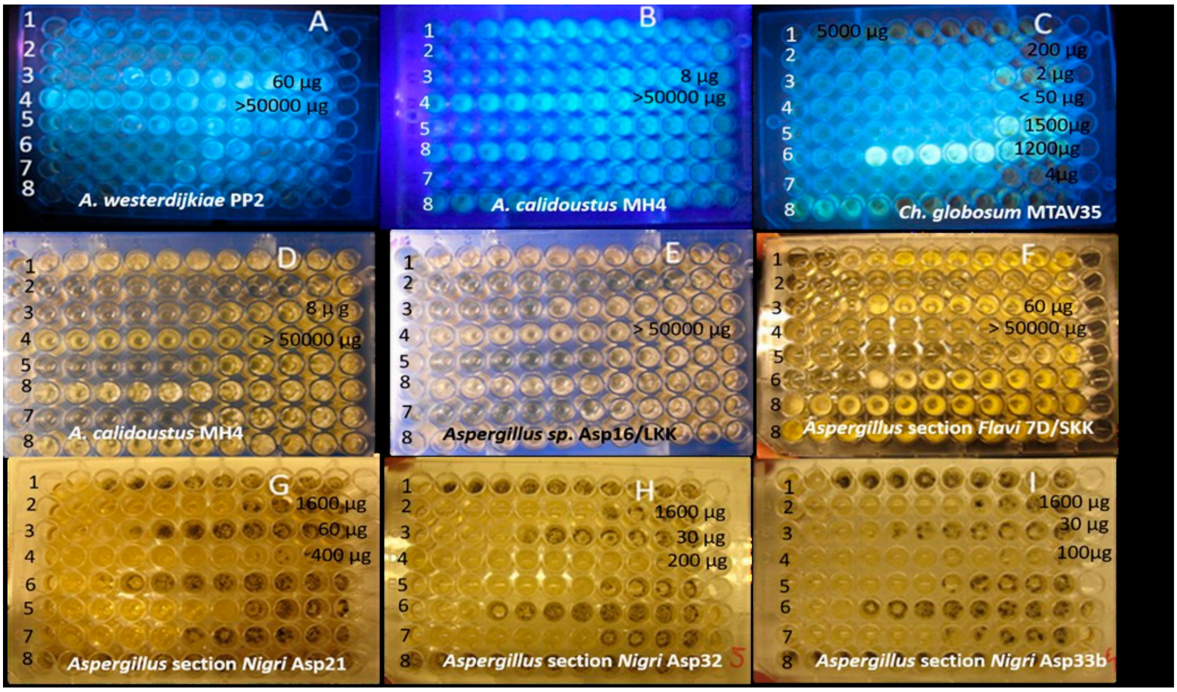

Figure S5. Resistance to biocides and Genapol visible as fluorescence emission (upper row), turbidimetry (middle row) and resporulation (bottom row). The row number indicates the biocide in two-fold dilutions from left to right as follow: Borax (1), Boracol (2), PHMB (3), Genapol (4), phenoxyethanol (5), chloramine (6), triclosan (7) and negative control (8). In the plates, in the upper row the germination of conidia is indicated by blue or blue/green fluorescence (in panel C the fluorescence is covered by new ascospores in rows 1, 2, 7, and 8). The plates in the middle row show the resistance to the exposing chemicals as turbidity, the lower row shows the resistance as resporulation of new black conidia. The plates were inspected after incubations at 28 °C for 2 d (Plates A, B, D, and E), and 10 d (Plates C, F, G, H, and I).

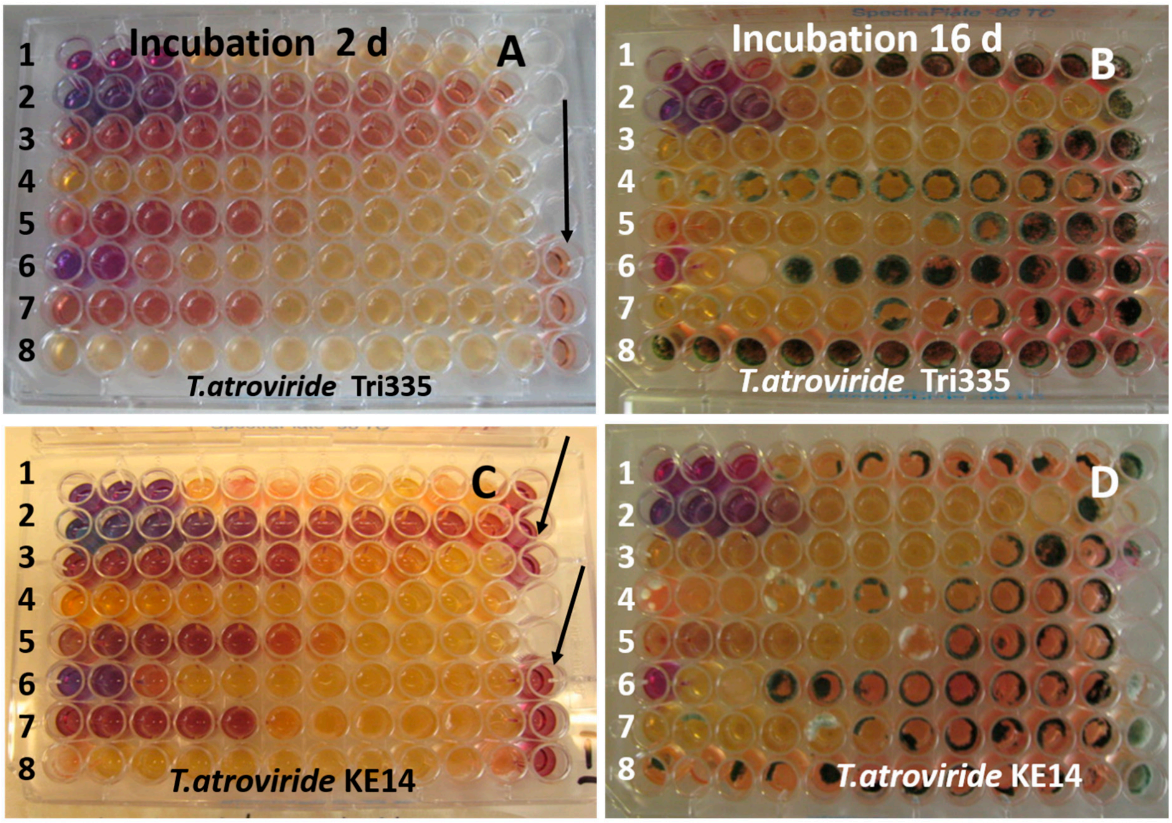

Figure S6. Resistance of biocides and Genapol to *Trichoderma atroviride* strains estimated as resazurin reduction (Panels A and C) and resporulation (B and D). The row number indicates the biocide in two-fold dilutions from left to right as follow: Borax (1), Boracol (2), PHMB (3), Genapol (4), phenoxyethanol (5), chloramine (6), triclosan (7) and negative control (8). Black arrows indicate wells filled with the medium only. Resazurin added after 2 d of incubation was metabolised by viable moulds to colourless dihydroresorufin. After 16 d of incubation, resporulation is visible by wells filled with green conidia.
